# Nutritional supplementation of vitamin A and health-related outcomes in patients with multiple sclerosis: A protocol for a systematic review and meta-analysis of randomized clinical trials

**DOI:** 10.1097/MD.0000000000016043

**Published:** 2019-06-21

**Authors:** Ana Clara de França Nunes, Grasiela Piuvezam

**Affiliations:** Department of Public Health, Federal University of Rio Grande do Norte, Natal/RN, Brazil.

**Keywords:** autoimmune diseases, inflammation, multiple sclerosis, nutraceuticals, vitamin A

## Abstract

**Background:**

Multiple sclerosis (MS) is a chronic immune mediated disease which affects the central nervous system (CNS), having a substantial financial, functional, and quality of life (QOL) impact on these people. The vitamin A supplementation has been studied as a therapeutic possibility for in MS. Therefore, the objective of this protocol is to build an outline for a future systematic review, which will provide up-to-date available evidence about the clinical impact of nutritional supplementation of vitamin A in the outcomes related to the symptoms in patients with this pathology.

**Methods:**

The search will be performed in the following databases: PubMed, Embase, Scopus, cinahl, Scielo, Web of Science, the Cochrane Library and Science Direct, randomized clinical trials published until May 2019 that evaluate the relationship of the supplementation of vitamin A and health-related outcomes in patients with MS will be included. Preferred Reporting Items for Systematic review and Meta-Analysis Protocols (PRISMA-P) will be used to outline the protocol, and PRISMA to the systematic review. Undergraduate handbook of quality of evidence and strength of recommendations for decision making in health (GRADE) will be used to assess the quality of evidence and the strength of the recommendation, and the JADAD scale to assess the internal validity of selected studies. For the extraction of all the data found a database in Microsoft Excel will be created. For the summary of the findings the Cochrane Collaboration Handbook recommendations will be used, and for the meta-analysis standard statistical techniques the RevMan software will be used.

**Results:**

In this study, we hope to find a considerable number of articles presenting evidence about the effectiveness of vitamin A supplementation in patients with MS.

**Conclusion:**

Currently, many lines of evidence have been produced when it comes to the use of food supplements. This systematic review proposal might provide recent, important, and trusted information for better treatment of patients.

**Record of systematic review:**

This review was recorded in the International Register of Prospective Systematic Reviews (PROSPERO) on the January 30, 2019 (registration: CRD42019121757). Available at: http://www.crd.york.ac.uk/PROSPERO/display_record.php?ID=CRD42019121757.

## 1 Introduction

Multiple sclerosis (MS) is a chronic immune mediated disease affecting the central nervous system (CNS), it strongly suggests that T cells and B cells are selectively recruited by specific target antigens (probably autoantigens) that are expressed only in the CNS.^[1]^ The disease predominantly affects individuals at the beginning of adulthood; the mean age for the onset of MS is 30 years old, having a substantial functional, financial, and quality of life (QOL) impact on these people.^[2]^

The overall prevalence of MS was estimated to be 2.3 million people in 2013, an increase of 200,000 people in the last 5 years, although it is likely to be underestimated, given the relative lack of data on large populations, including India and China. Moreover, it is known that the proportion of women is higher (2:1).^[3]^ Environmental, genetic, and epigenetic factors have a causal role in MS and potentially interact with the modifiable risk factors.^[4]^

While some people with MS live with little disability during life, around 60% may become unable to walk without assistance about 20 years after the onset of the disease. This represents important implications for the QOL of people with MS, their relatives and friends, and also the high cost it poses on society.^[3]^

In addition to conventional medicine, there is a growing public and scientific interest in incorporating strategies to optimize the care of MS as part of an integrative model of medicine and public health. Many of these interventions are rooted in a comprehensive health and welfare approach that focuses on physical and emotional well-being including diet, dietary supplements, physical activity, stress control, and smoking cessation.^[5]^

These alternative therapies with a low cost can bring countless benefits to patients, their care network and the public health system. Moreover, it is of paramount importance that there is a strengthened network of primary health care for the identification of the disease in its initial stage, and the agile and adequate referral for specialized care aimed at a better therapeutic outcome and prognosis of cases.^[6]^

Among the environmental factors that can influence the MS course are food and lifestyle. In this sense, it is possible to affirm that specific nutritional factors can have anti-inflammatory effects, as is the case of antioxidant vitamins.^[7]^

Vitamin A plays a role in various aspects of immune function including the innate and adaptive immune system and may be linked to the development of regulatory T cells,^[8]^ which may be relevant in the pathogenesis of MS.^[9]^ Retinoic acid modulates the balance between auxiliary (TH) 1, TH2, TH17, and T (Tregs) regulatory cells, the function of dendritic cells, B cells, and microglia,^[10,11]^ in addition to binding to specific intranuclear receptors (the retinoic acid receptor [RAR] and retinoid receptor X [RXR]), which regulate the transcription of vitamin A-responsive genes including genes related to re-myelination.^[12]^

Vitamin A supplementation has been studied as a therapeutic possibility for MS^[13]^ for its proven benefits at a cellular level.^[14,15]^ Moreover, it is known that micronutrients supplementation is a safe, inexpensive and convenient method to perform.^[16]^

Therefore, based on the evidence demonstrating the possible benefits of vitamin A nutritional supplementation in the pathogenesis of multiple sclerosis, it is imperative to establish recommendations for it to be used from primary health care to research in this area. Therefore, the objective of this systematic review and meta-analysis will be to provide the best available evidence on the clinical impact of vitamin A nutritional supplementation on health-related outcomes in patients with multiple sclerosis.

## 2 Methods and analysis

### 2.1 Protocol and registration

This review was recorded in the International Prospective Register of Systematic Reviews (PROSPERO) on the 30th of January 2019 (Record: CRD42019121757). Available at: http://www.crd.york.ac.uk/PROSPERO/display_record.php?ID=CRD42019121757.

The study in question is a protocol for a systematic review, so it was not necessary for an ethics committee or an institutional review board approved it.

### 2.2 Analysis plan

**Figure 1:**
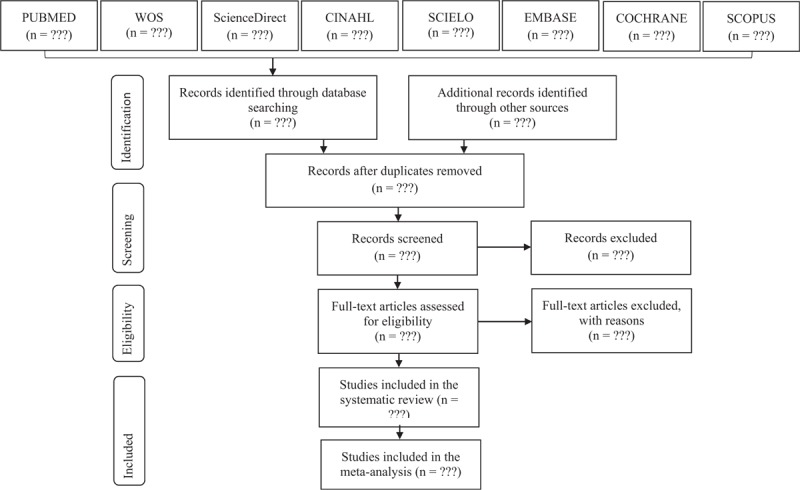
PRISMA flow diagram. PRISMA = Preferred Reporting Items for Systematic reviews and Meta-Analyse.

This protocol was developed in accordance with the guidelines described by the Preferred Reporting Items for Systematic review and Meta-Analysis Protocols (PRISMA-P),^[17]^ and for the design of the systematic review the PRISMA^[18]^ Checklist will be used, a checklist of 27 items and a selection flow diagram (Fig. 1) of four-phase articles, which aims to improve the quality of the data from the systematic review and meta-analysis.

### 2.3 Selection criteria

#### 2.3.1 Types of studies

Only randomized controlled trials (RCTs) will be included in this systematic review, in any language. The choice to include only RCTs was because this type of study offers a better quality of evidence, due to the methodological process used.

#### 2.3.2 Types of participants

We will accept adult patients diagnosed with MS of any sex and race. Studies containing patients with other complicating diseases will not be included.

#### 2.3.3 Types of interventions

We will include studies that have used the nutritional supplementation of vitamin A as an experimental treatment to improve the symptoms of MS regardless of its treatment form, dosage, frequency, and duration.

#### 2.3.4 Types of outcomes

We will include studies that present at least one of these outcomes:

##### 2.3.4.1 Primary

The improvement or stagnation of the disease progression (according to Kurtzke expanded disability status scale—EDSS), and/or the improvement or stagnation of the disease activity (measured by magnetic resonance imaging [MRI]), and/or the improvement of fatigue and/or the improvement of depressive symptoms.

##### 2.3.4.2 Secondary

Improvement of the relapse rate, and/or an improvement in QOL. In addition, only studies in which patients are classified in the extended disability status scale of Kurtzke (EDSS) from 0 to 5 were included and patients in concomitant drug treatment with supplementation.

### 2.4 Data collection and analysis

#### 2.4.1 Search strategy

A broad electronic search strategy will be employed, including the following databases: PubMed, Excerpta Medica Database (Embase), Scopus, Cinahl, Scielo, Web of Science, Cochrane Central Register of Controlled Trials (CENTRAL) and Science Direct to identify articles published by May 2019. The search strategy will include the following medical subject headings (MeSH): “Vitamin A,” “Tretinoin,” “Retinol palmitate,” “Carotenoids,” “Multiple sclerosis,” and “MS.”

#### 2.4.2 Searches of other resources

The references of the included manuscripts will also be reviewed to potentially identify any missed attempts. To identify any unpublished studies and evaluate the publication bias, we will also look at ClinicalTrials.gov and ensaiosclinicos.gov.br for registered clinical trials of vitamin A nutritional supplementation in patients with MS.

#### 2.4.3 Study selection

The articles will be imported to Mendeley (1.17.11), and the initial evaluation of the studies will be done through the title and the abstract using the Search strategies (Table 1), following the inclusion and exclusion criteria described below by 2 independent researchers (KF and RP). If any summary is not provided using the search strategy above, the full text of the manuscript will be revised and evaluated. If the 2 independent researchers disagree on whether a specific study meets the inclusion criteria for the review, a third researcher (AC) will decide whether or not to include the study. The full text of all the studies considered eligible for inclusion will then be revised by all the researchers for analysis.

**Table 1:**
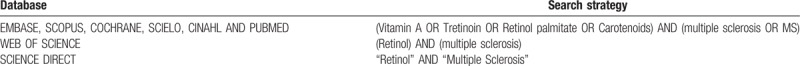
Search strategies for each database.

### 2.5 Evidence quality assessment (GRADE)

Two independent authors (AC and KF) will evaluate the quality of evidence and the strength of the recommendations provided by the selected studies included. For this the quality of evidence undergraduate manual and the strength of the recommendation for health decision Making (GRADE)^[19]^ will be used, which classifies the quality of evidence into 4 levels: high, moderate, low, and very low, and the strength of evidence into 2 levels: strong or weak.

### 2.6 Evaluation of methodological quality (JADAD scale)

Two independent authors (AC and RP) will evaluate the internal validity of all the eligible RCTs. For this, the Jadad scale^[20]^ will be used, which consists of a list of 5 questions, evaluating 3 aspects of the studies: randomization, blinding and losses in the conduction of the RCTs, resulting in a score ranging from 0 to 5, studies with a score ≤3 are considered at a high risk of bias.

### 2.7 Data collection and analysis

#### 2.7.1 Selection of literature

The extraction of all the data will be done in a standardised way, creating a database using Microsoft Excel (version 2013) by 2 independent authors (AC and KF). The following information will be presented in this database: first author, year of study, study site, population (quantity and characteristics related to age and sex), supplementation dosage, follow-up time, results, and other relevant information. The entire database will be reviewed by 2 researchers to ensure the accuracy of the information. For any relevant missing data, we will contact the authors of the study. If we fail to receive any necessary information, the data will be excluded from our analysis and will be addressed in the Discussion section.

#### 2.7.2 Data extraction

The data extracted from the included articles will be presented descriptively, and the effect sizes will be calculated based on the recommendations of the Cochrane Collaboration Handbook (5.1).

#### 2.7.3 Measures of treatment effect

We will use the Rev Man Analyses statistical package in the Review manager (RevMan) [computer program]. Version 5.3. Copenhagen, Denmark. The continuous outcome data will be expressed as mean differences, while the dichotomous outcomes will be derived from the odds ratio (OR) and 95% confidence interval (CI) for each study. The heterogeneity between the trial results will be evaluated using a standard chi-square test with a significance level of 0.05.

#### 2.7.4 Assessment of heterogeneity

To evaluate the heterogeneity, we plan to compute the chi-square statistic, which is a quantitative measure of inconsistency between the studies. A value of 0% indicates that there is no heterogeneity observed, whereas the values of *I*^2^ of 50% indicate a substantial level of heterogeneity.

#### 2.7.5 Data synthesis

In the development of meta-analysis, standard statistical techniques will be used. The heterogeneity between the results of the study will be evaluated using a standard *I*^2^ test with a significance level of *P* = .1. If there is heterogeneity (*I*^2^ = 75%), a random effects model will be used to combine the tests to calculate the relative risk (RR) and 95% CI, using the DerSimonian-Laird algorithm in a meta for R package. If the tests for heterogeneity have no significant meaning (*I*^2^ ≤50%), the fixed effect model will be used.

#### 2.7.6 Assessment of reporting bias

If possible, funnel plots will also be used to evaluate the presence of potential report biases, and a linear regression approach will be used to evaluate the asymmetry of the funnel chart.

#### 2.7.7 Subgroup analysis

A subgroup analysis will be performed when the preliminary result of the data synthesis indicates the subgroup is needed, based on the different characteristics, types of intervention, research scenario, and result tools.

## 3 Discussion

The objective will be to evaluate, through a systematic review, the clinical impact of vitamin A nutritional supplementation on the health-related outcomes in patients with MS, taking into account that the reduction in the irreversible accumulation of neurological disability is one of the main targets in the care of people with this pathology, and that the identification of possible improvements in the course of the disease is essential for better patient care.^[21]^

Currently, many lines of evidence have been provided regarding the use of dietary supplements in MS, with vitamin D being the main target. In this case, epidemiological data, in particular, preclinical investigations, animal studies, and association studies on vitamin D supplementation and disease activity suggest that higher serum vitamin D concentrations are beneficial in terms of the risk of developing MS, as well as for the disease already established.^[16]^ The study conducted by Røsjø et al^[22]^ found that an increase in levels of 25 (OH)D (vitamin D) is positively associated with an increase in retinol (vitamin A) levels.

In the case of vitamin A (retinol), the evidence suggests that inadequate levels result in the organism's inability to maintain the normal balance of the T-cell subgroups,^[23]^ as well as a negative correlation between serum vitamin A and the development of the disease; due to the fact that the plasmatic level of vitamin A is lower in patients with MS.^[24,25]^ A cohort study suggested an inverse association of vitamin A levels in serum and the activity of relapsing-remitting MS by means of magnetic resonance imaging.^[26]^

It is worth mentioning that in an in vitro model using pluripotent cerebral microvascular endothelial cells derived from stem cells, retinoic acid (RA) reinforced the properties of the blood–brain barrier (BBB),^[27]^ suggesting that RA could block cell traffic through the BBB. These observations predict that retinol may have beneficial immunological effects on the activity of MS and sustain the idea that lower levels of serum retinol are associated with a greater loss of brain volume that results in more severe disability in the future.^[28]^

Another study, also at the molecular level (a systematic review), involving the supplementation of patients with autoimmune diseases (MS and atherosclerosis), all with applications of 25,000 IU/d of retinyl palmitate, presented as the main hypothesis that the 25,000 IU/d dosage of retinyl palmitate for 3 to 6 months may increase serum retinoic acid (RA) levels in patients and significantly increase the expression of the RA receptor gene, thus suggesting that vitamin A supplementation can improve the level of serum cytokines and the clinical signs of autoimmune disease.^[29]^

It is suggested, therefore, that vitamin A supplementation can be a promising strategy for the management and/or prevention of multiple sclerosis,^[30]^ being beneficial to relieve inflammation and useful to protect the brain, as well as possibly being able to benefit patients in the degenerative phase. In addition, vitamin A supplementation is recommended to be a part of the MS therapeutic program.^[31]^

With regard to QOL, it is known that people with multiple sclerosis face a multitude of physical, mental, and emotional challenges on a daily basis,^[32,33]^ presenting not only a significantly worse QOL than the general population but also compared with those diagnosed with other long-lasting diseases such as epilepsy, diabetes, rheumatoid arthritis, and irritable bowel disease.^[34]^ The most common symptoms of MS are sensory and motor problems; however, the experience of each person with MS is different, which justifies a large number of symptoms that may be present at the onset of the disease; some of these symptoms are less easily perceived, for example, as is the case of fatigue.^[3]^

Depression is a symptom frequently observed in these patients, which leads to estimates of suicide rates in people with MS as being quite high, indicating an increase of approximately 2 times in relation to the general population, with attempts and suicidal ideations present in this group^[35]^; therefore, untreated or poorly managed symptoms can lead to serious and potentially fatal complications.^[36]^

From this, the proposed systematic review may provide important information for better treatment for patients with MS. The results have the potential to inform national and international guidelines on the importance of maintaining good serum levels of vitamin A in this population. The review will also help to highlight areas that require the need for further research on the subject.

## Author contributions

**Conceptualization:** Ana Clara de França Nunes, Grasiela Piuvezam.

**Data curation:** Ana Clara de França Nunes, Grasiela Piuvezam.

**Formal analysis:** Grasiela Piuvezam.

**Funding acquisition:** Ana Clara de França Nunes, Grasiela Piuvezam.

**Investigation:** Ana Clara de França Nunes.

**Methodology:** Ana Clara de França Nunes, Grasiela Piuvezam.

**Project administration:** Ana Clara de França Nunes, Grasiela Piuvezam.

**Resources:** Ana Clara de França Nunes, Grasiela Piuvezam.

**Software:** Ana Clara de França Nunes.

**Supervision:** Grasiela Piuvezam.

**Validation:** Ana Clara de França Nunes.

**Visualization:** Ana Clara de França Nunes.

**Writing – original draft:** Ana Clara de França Nunes.

**Writing – review & editing:** Grasiela Piuvezam.
